# Early *Brassica* Crops Responses to Salinity Stress: A Comparative Analysis Between Chinese Cabbage, White Cabbage, and Kale

**DOI:** 10.3389/fpls.2019.00450

**Published:** 2019-04-11

**Authors:** Iva Pavlović, Selma Mlinarić, Danuše Tarkowská, Jana Oklestkova, Ondřej Novák, Hrvoje Lepeduš, Valerija Vujčić Bok, Sandra Radić Brkanac, Miroslav Strnad, Branka Salopek-Sondi

**Affiliations:** ^1^Department of Molecular Biology, Ruđer Bošković Institute, Zagreb, Croatia; ^2^Laboratory of Growth Regulators, Institute of Experimental Botany, The Czech Academy of Sciences, Palacký University, Olomouc, Czechia; ^3^Department of Biology, Josip Juraj Strossmayer University of Osijek, Osijek, Croatia; ^4^Faculty of Humanities and Social Sciences, Josip Juraj Strossmayer University of Osijek, Osijek, Croatia; ^5^Faculty of Dental Medicine and Health, Josip Juraj Strossmayer University of Osijek, Osijek, Croatia; ^6^Division of Botany, Department of Biology, Faculty of Science, University of Zagreb, Zagreb, Croatia

**Keywords:** Chinese cabbage, kale, salinity stress, photosynthetic performance, stress hormones, brassinosteroids, tolerance, white cabbage

## Abstract

Soil salinity is severely affecting crop productivity in many countries, particularly in the Mediterranean area. To evaluate early plant responses to increased salinity and characterize tolerance markers, three important *Brassica* crops – Chinese cabbage (*Brassica rapa* ssp. *pekinensis*), white cabbage (*B. oleracea* var. *capitata*) and kale (*B. oleracea* var. *acephala*) were subjected to short-term (24 h) salt stress by exposing them to NaCl at concentrations of 50, 100, or 200 mM. Physiological (root growth, photosynthetic performance parameters, and Na^+^/K^+^ ratio) and biochemical parameters (proline content and lipid peroxidation as indicated by malondialdehyde, MDA, levels) in the plants’ roots and leaves were then measured. Photosynthetic parameters such as the total performance index PI_total_ (describing the overall efficiency of PSI, PSII and the intersystem electron transport chain) appeared to be the most salinity-sensitive parameter and informative stress marker. This parameter was decreased more strongly in Chinese cabbage than in white cabbage and kale. It indicated that salinity reduced the capacity of the photosynthetic system for efficient energy conversion, particularly in Chinese cabbage. In parallel with the photosynthetic impairments, the Na^+^/K^+^ ratio was highest in Chinese cabbage leaves and lowest in kale leaves while kale root is able to keep high Na^+^/K^+^ ratio without a significant increase in MDA. Thus Na^+^/K^+^ ratio, high in root and low in leaves accompanying with low MDA level is an informative marker of salinity tolerance. The crops’ tolerance was positively correlated with levels of the stress hormone abscisic acid (ABA) and negatively correlated with levels of jasmonic acid (JA), and jasmonoyl-L-isoleucine (JA-Ile). Furthermore, salinity induced contrasting changes in levels of the growth-promoting hormones brassinosteroids (BRs). The crop’s tolerance was positively correlated with levels of BR precursor typhasterol while negatively with the active BR brassinolide. Principal Component Analysis revealed correlations in observed changes in phytohormones, biochemical, and physiological parameters. Overall, the results show that kale is the most tolerant of the three species and Chinese cabbage the most sensitive to salt stress, and provide holistic indications of the spectrum of tolerance mechanisms involved.

## Introduction

Global warming and associated climate changes are imposing severe abiotic stresses that are seriously impairing crop yields and quality in many affected areas. One of these stresses (exacerbated by various human activities) is soil salinity. Over 7% of the world’s total land and approximately 20% of irrigated land is affected by high salinity. The problem is particularly severe in the Mediterranean, semi-arid and arid areas (Zhang et al., 2014; Munns and Gilliham, 2015).

Plants’ growth rates and productivity depend on photosynthetic efficiency. Thus, it is highly important for them to adjust their photosynthetic apparatus in accordance with environmental stresses. Photosystem II (PSII) is the most sensitive part of the apparatus to salt stress (Kalaji et al., 2011; Jajoo, 2014; Oukarroum et al., 2015). However, salinity stress has complex effects on photosynthetic activity, depending on the species or cultivar, duration of the stress and salt concentration. Low salt concentrations usually induce adaptations of photosynthetic activity, overall connectivity of the photosystem units and functional antenna size that maintain or even increase photosynthetic efficiency (Mehta et al., 2010; Dabrowski et al., 2016). In contrast, higher salinity usually causes photoinhibition of both photosystems (PSII and PSI), inhibits overall electron transport chain activity and increases non-photochemical quenching (Jajoo, 2014).

Salt concentrations exceeding taxa-specific thresholds induce three kinds of interacting stresses that are collectively called salinity stress. These are ionic stress caused by toxic concentrations of ions (mainly Na^+^), osmotic stress caused by associated reductions in water uptake, and oxidative stress mainly driven by increases in levels of reactive oxygen species (ROS) (Liang et al., 2018). Energy-efficient osmotic adjustment of cell turgor by the accumulation of Na^+^ and Cl^-^ in leaves and roots is a characteristic response of salt-tolerant species and halophytes. In addition, maintaining low Na^+^ (and Cl^-^) concentrations in the cytoplasm of cells, optimizing the concentration of essential K^+^ and efficient sequestration of salt ions in vacuoles are key elements of “tissue tolerance” (Munns et al., 2016). In fact, Na^+^ competing with the binding site of K^+^ on proteins e.g., channels and enzymes, is the main reason for Na^+^ toxicity (Almeida et al., 2017). High Na^+^ concentrations in cellular cytoplasm can inactivate enzymes and metabolic processes. Furthermore, the accumulation of Na^+^ in leaves can inhibit photosynthesis if ions are not adequately compartmentalized at the cellular or subcellular level (Julkowska and Testerink, 2015). Moreover, more salt-sensitive species respond by *de novo* synthesis of osmolytes and allocation of growth assimilates to osmotic adjustment, with consequent increases in energy costs and reductions in growth rates (Munns et al., 2016). In addition, the oxidative stress associated with high salinity necessitates induction of antioxidant mechanisms to detoxify ROS, as inefficient ROS removal can result in damage to essential macromolecules (proteins, lipids, and nucleotides) and cell death.

One of the key mediators and modulators of plants’ responses to all environmental factors are phytohormones, the main plant signaling molecules (Verma et al., 2016; Raja et al., 2017). Complex networks of interacting phytohormones [abscisic acid (ABA), salicylic acid, jasmonates (JAs), brassinosteroids (BRs), cytokinins, ethylene, auxins, and gibberellins] play crucial roles in plants’ physiological responses and adaptation to salinity stress (Fahad et al., 2015). One of these phytohormones, ABA, is a key mediator of osmotic stress responses (Fahad et al., 2015). ABA regulates transpiration rates and maintains cellular turgor by controlling stomatal opening and closure (in concert with other phytohormones), induces osmoprotectant accumulation, activates ROS detoxification mechanisms, and modifies ion transport (Finkelstein, 2013; Fahad et al., 2015). JAs also modulate plants’ growth, development and abiotic stress responses (Ahmad et al., 2016). They play key roles, *inter alia*, in the development of embryos, seedlings and floral organs, seed germination, growth inhibition, and senescence. JAs also participate interactively with other phytohormones in crops’ performance affecting adaptations to changes in environmental conditions (Per et al., 2018). *Inter alia*, crosstalk between ABA and JAs regulates stomatal closure and transpiration under drought conditions, and in saline conditions, JAs are involved in the control of uptake of sodium ions (Riemann et al., 2015). BRs also have been recently reported roles in plant tolerance to diverse stress factors (e.g., salt, drought, and temperature stresses) (Jiroutova et al., 2018). BRs participate in the control of cell cycling and growth, modification of cell wall architecture, and adjustment of membrane systems. Moreover, they contribute to the maintenance of cells’ redox systems and the regulation of stomatal aperture in drought and salinity responses (Sharma et al., 2017).

To meet problems caused by salinity, knowledge of major crops’ tolerance levels and mechanisms is clearly important. Currently, most *Brassica* crop species are classified as moderately salt tolerant. However, the amphidiploid species *B. juncea*, *B. napus*, and *B. carinata* reportedly have a somewhat higher tolerance than the diploids *B. oleracea*, *B. nigra*, and *B. rapa* (Purty et al., 2008). The reasons for this are uncertain, and there is little knowledge of tolerance mechanisms in the family. To assist efforts to elucidate mechanisms responsible for salt tolerance in Brassicaceae, three *Brassica* crops with global economic importance were selected for this study: white cabbage (*Brassica oleracea* var. *capitata*), kale (*B. oleracea* var. *acephala*), and Chinese cabbage (*B. rapa* L. *pekinesis*). Given the complexity of tolerance mechanisms, a holistic approach was chosen in efforts to elucidate salinity responses of the three *Brassica* crops.

We hypothesized that early, initial salinity responses may be essential for long-term salinity tolerance and provide insights into the involvement of different defense mechanisms. Moreover, we hypothesized that fine tuning of hormonal status plays major roles in early salt stress responses and further adaptation of the selected brassicas. We first determined their sensitivity/tolerance to applied salinity stresses by root-growth bioassays and biomass production. Then photosynthetic parameters of hydroponically grown plants were measured following short-term salinity stress. In parallel, biochemical parameters involved in responses to oxidative stress (the level of lipid peroxidation), osmotic stress (proline accumulation), and ionic stress (Na^+^/K^+^ ratio) were measured in leaves and roots of treated plants in comparison to corresponding controls. We also measured changes in levels of ABA, JAs, and BRs to explore their roles in the regulation of salinity responses in the selected crops. Finally, Principal Component Analysis (PCA) of acquired data enabled us to draw correlations regarding traits of the crops and identify key contributors to differences in their salinity responses.

## Materials and Methods

### Plant Material and Experimental Conditions

Seeds of Chinese cabbage (*B. rapa* L. ssp. *pekinensis* (Lour.) Hanelt cv. Cantonner Witkrop), white cabbage (*B. oleracea* var. *capitata* cv. Varaždinski) and kale (*B. oleracea* var. *acephala* cv. IJK9) were purchased from ISP International Seed Processing GmbH, Quedlinburg, Germany, the Agricultural Advisory Service of Varaždin Region, Croatia, and Institute for Adriatic Crops and Karst Reclamation, Split, Croatia, respectively.

The level of salinity tolerance/sensitivity of the selected *Brassica* crops was determined using the root-growth bioassay described by Pavlović et al. (2018a). The effect of NaCl on the seedlings biomass production was evaluated after 7 days of treatments in comparison to the corresponding controls.

For hydroponic growth, seeds were germinated on 1% agar plates and then several-day-old seedlings were placed in a home-made hydroponic growth system supplying commercially available nutrient solutions (Flora Series and GHE Hydroponics) according to the manufacturers’ instructions. Plants were grown in 5.5 L dark pots in a growing chamber at 21°C, with 16/8 h light (115 μmol m^-2^ s^-1^)/dark cycles.

At the four fully developed leaf stage (after 3–4 weeks, depending on the cultivar) sets of the plants were subjected to salinity stress, by incrementally increasing concentrations of NaCl for 25 mM and 50 mM in the nutrient solution (in order to avoid shock). Salinization was performed at 2 h intervals to final concentrations as follows: 50 mM NaCl (25 mM/two steps), 100 mM Na (25 mM/four steps), and 200 mM NaCl (25 mM/four steps and 50 mM/two steps), respectively. The nutrient solution of controls of each species remained unchanged. After 24 h of exposure to the final salt concentrations, the plants were harvested together with controls. The experiment was performed with four biological replicates, each consisting of eight plants from a pot, unless otherwise stated.

Plant material for biochemical analysis was stored at -80°C until use while material for hormonal profiling and determination of ion contents was freeze-dried until analysis. Photosynthesis measurements (fast chlorophyll *a* fluorescence kinetics) were performed on leaves *in vivo*, as described below.

### Fast Chlorophyll *a* Fluorescence Kinetics

Fast chlorophyll *a* fluorescence kinetics were measured on 18 randomly selected, dark-adapted leaves of each *Brassica* cultivar using a Handy-PEA fluorimeter (Hansatech, United Kingdom). After 30 min of dark adaptation, the leaves were exposed to a pulse of saturating red light (3200 μmol m^-2^ s^-1^, peak at 650 nm). OJIP transients were measured by recording data from 50 μs (F_0_) to 1 s (F_m_). Data extrapolated from the acquired OJIP curves were subjected to JIP-tests to calculate parameters according to Strasser et al. (2000). Calculations are shown in Supplementary Table S1.

The OJIP transients were double-normalized between O (50 μs) and P steps and presented as relative variable fluorescence, W_OP_ = (F_t_-F_0_)/(F_P_-F_0_). Fluorescence data were plotted on a logarithmic time scale, and the O, J, I, and P steps were marked in plots. Normalization between O and K (300 μs) steps revealed L-band (150 μs) which was presented as variable fluorescence W_OK_ = (F_t_-F_0_)/(F_K_-F_0_) and then plotted with difference kinetics ΔW_OK_ = W_OK_-(W_OK_)_ref_. Normalization between O and J (2 ms) steps revealed K-band, presented as variable fluorescence W_OJ_ = (F_t_-F_0_)/(F_J_-F_0_) and plotted with difference kinetics ΔW_OJ_ = W_OJ_-(W_OJ_)_ref_. Values measured in control plants were used as referent value (W_OK_)_ref_ (Strasser et al., 2004; Yusuf et al., 2010).

### Biochemical Stress Parameters

Levels of MDA and proline were determined spectrophotometrically using previously reported methods (Radić et al., 2009). Plant material for determination of MDA levels (250 mg of fresh weight of leaves and roots) was extracted in 2 ml of potassium phosphate buffer (50 mM, pH 7.0, 0.1 mM EDTA) with the addition of polyvinylpolypyrrolidone (PVPP). MDA levels in the extracts were estimated by MDA reaction with thiobarbituric acid, subtracting the absorbance at 600 nm from the absorbance at 532 nm, and using an extinction coefficient of 155 mM^-1^ cm^-1^.

Proline was extracted from 100 mg samples (FW) in 1.5 ml of 3% sulfosalicylic acid and determined spectrophotometrically at 520 nm using ninhydrin. Absorbance values were adjusted using a calibration curve constructed with L-proline as a standard and the results were expressed in nmol proline per g FW.

A Specord 40 spectrophotometer (Analytik Jena, Jena, Germany) was used for all absorbance measurements, with four replicates per assay.

### Contents of Sodium and Potassium Ions

Contents of sodium and potassium ions in roots and leaves of the *Brassica* crops were measured by high-resolution inductively coupled plasma mass spectrometry, using a Thermo Fisher Scientific HRICP-MS Element 2 instrument (Thermo Fisher Scientific, Bremen, Germany) equipped with an ESI-a SC-2 DX FAST autosampler (Elemental Scientific, United States) using indium as an internal standard. Typical instrumental conditions and measurement parameters used throughout the work have been previously reported (Fiket et al., 2016). Before analysis, powdered lyophilized tissue samples were subjected to microwave-assisted acidic digestion in HNO_3_/HF (60:1, v/v) using a Multiwave 3000 (Anton Paar, Graz, Austria) at 1400 W.

### Hormonal Profiling

#### Stress-Related Hormones

The stress-related phytohormones, JAs (jasmonic acid, JA, and jasmonoyl-L-isoleucine, JA-Ile) and ABA were determined as previously described (Floková et al., 2014) with minor modifications. Briefly, lyophilized samples (5 mg dry weight, DW) were homogenized with a MM 301 vibration mill (Retsch GmbH, Haan, Germany), extracted in 1 mL 50 mM sodium phosphate buffer (pH 7.0) containing 1% sodium diethyldithiocarbamate and stable isotope-labeled internal standards (10 pmol [^2^H_6_]JA, 10 pmol [^2^H_6_]ABA and 0.1 pmol [^2^H_2_]JA-Ile; OlchemIm, Olomouc, Czechia) then alkalized by adding 1 mL of 5% NH_4_OH/H_2_O (*v*/*v*). The resulting solution was purified by passage through a mixed-mode anion exchange column (Oasis^^®^^ MAX column, 1 cc/30 mg, Waters, Milford, MA, United States) conditioned with 100% MeOH and equilibrated with H_2_O and 5% NH_4_OH (1 ml of each solution). After sample loading, the column was washed with 2 mL 5% NH_4_OH followed by 2 mL 100% MeOH, then the acidic phytohormones were eluted using 2 mL 2% HCOOH in 100% MeOH (*v*/*v*). The samples were evaporated to dryness under a stream of nitrogen and stored in a freezer at -20°C until analysis.

#### Brassinosteroids

Samples’ BR contents were analyzed as previously described (Oklestkova et al., 2017) with a few modifications. Briefly, lyophilized samples (40 mg DW) were homogenized to a fine consistency using 3 mm zirconium oxide beads and an MM 301 vibration mill at a frequency of 30 Hz for 3 min (Retsch, Haan, Germany). The samples were then extracted overnight with stirring at 4°C using a benchtop laboratory rotator (Stuart SB3; Bibby Scientific, Cole-Parmer, Staffordshire, United Kingdom) after adding 1 mL of ice-cold 60% acetonitrile and 30 pmol of [^2^H_3_]brassinolide (BL), [^2^H_3_]castasterone (CS), [^2^H_3_]typhasterol (TY), [^2^H_3_]24-*epi*BL, [^2^H_3_]24-*epi*CS, [^2^H_3_]28-norBL, and [^2^H_3_]28-norCS (OlchemIm, Olomouc, Czech Republic) as internal standards. The samples were then centrifuged, purified using DPA-6S SPE columns (Supelco, Bellefonte, PA, USA) and evaporated to dryness *in vacuo*. They were then dissolved in 75 μl 100% MeOH with sonication, made up to 1 ml with PBS buffer (pH 7.2) then loaded on an immunoaffinity column (IAC) coated with anti-BR monoclonal antibodies. After washing the IAC with 9 mL H_2_O, BRs were eluted using 3 mL ice-cold MeOH (-20°C), evaporated *in vacuo* using a CentriVap^^®^^ acid-resistant benchtop concentrator (Labconco Corp., MO, USA) and stored at -20°C until analysis.

#### UHPLC-MS/MS Analysis

For ultra-high performance liquid chromatography-tandem mass spectrometry (UHPLC-MS/MS) analysis, all samples were dissolved in 40 μL of the mobile phase and analyzed using an Acquity UPLC System (Waters, Milford, MA, USA) coupled to a Xevo^TM^ TQ-S MS triple quadrupole mass spectrometer (Waters, MS Technologies, Manchester, United Kingdom). Stable isotope-labeled internal standards were used as references and concentrations of analytes were quantified by the isotope dilution method. Previously published instrument settings were used for profiling stress-related phytohormones (Floková et al., 2014) and BRs (Oklestkova et al., 2017). Four independent replicate analyses were performed for each hormone group if not stated otherwise.

### Statistical Analysis

Between-treatment differences in measured variables of each of the *Brassica* crops were evaluated using factorial analysis of variance (ANOVA) followed by the *post hoc* Tukey Honest Significant Difference (HSD) test. Differences were considered significant if *p* < 0.05. To minimize bias in comparisons of the varieties, which differed in salinity tolerance, all data were normalized to control values. Data presented in the text, figures, and tables are means ± standard deviation of four replicates (*n* = 4) for biochemical markers and phytohormones, and means ± standard deviation of 18 replicates (*n* = 18) for fluorescence measurements.

### Principal Component Analysis

Correlations among the measured physiological, biochemical, and hormonal parameters, treatments and responses of the *Brassica* crops were explored by PCA. PCA was performed using the correlation matrix of the average values of traits after standardization (autoscaling). Linear correlations among variables were determined by Pearson coefficients (*p* < 0.05). XLSTAT software (ver. 2017.01.40777) implemented in Microsoft Office Excel 2010 was used for all statistical procedures.

## Results

### Salinity Tolerance Evaluation by Root-Growth Bioassay

**FIGURE 1:**
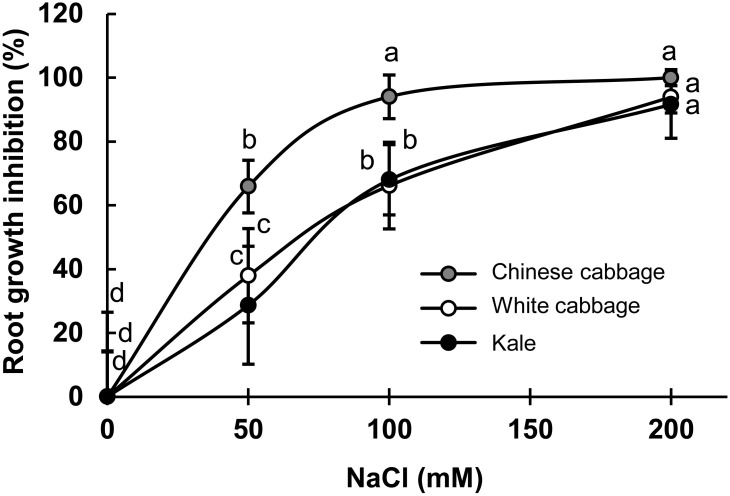
Dose-dependent root-growth inhibition of Chinese cabbage (*B. rapa*), white cabbage (*B. oleracea* var. *capitata*), and kale (*B. oleracea* var. *acephala*) exposed to indicated NaCl concentrations (0–200 mM), relative to salt-free controls (%). Presented data are means ± SD, *n* = 30. Points labeled with different letters differ significantly at *p* < 0.05.

The inhibition of root-growth during exposure to NaCl at 50–200 mM for 24 h was used as a quick, convenient bioassay to evaluate the salinity tolerance of the selected brassicas (Figure 1). As shown in Figure 1, the treatments caused dose-dependent root-growth inhibition in all three varieties, but most strongly in Chinese cabbage. Root-growth inhibition rates were similar in white cabbage and kale. Seedlings biomass production upon prolonged salinity stress (up to 7 days) showed statistically significant inhibition in Chinese cabbage and white cabbage at higher salt concentrations (100 and 200 mM NaCl), while there is no significant inhibition in biomass production in kale (Supplementary Figure S1 in the Supplementary File).

### Salinity Effects on Photosynthetic Performance

**FIGURE 2:**
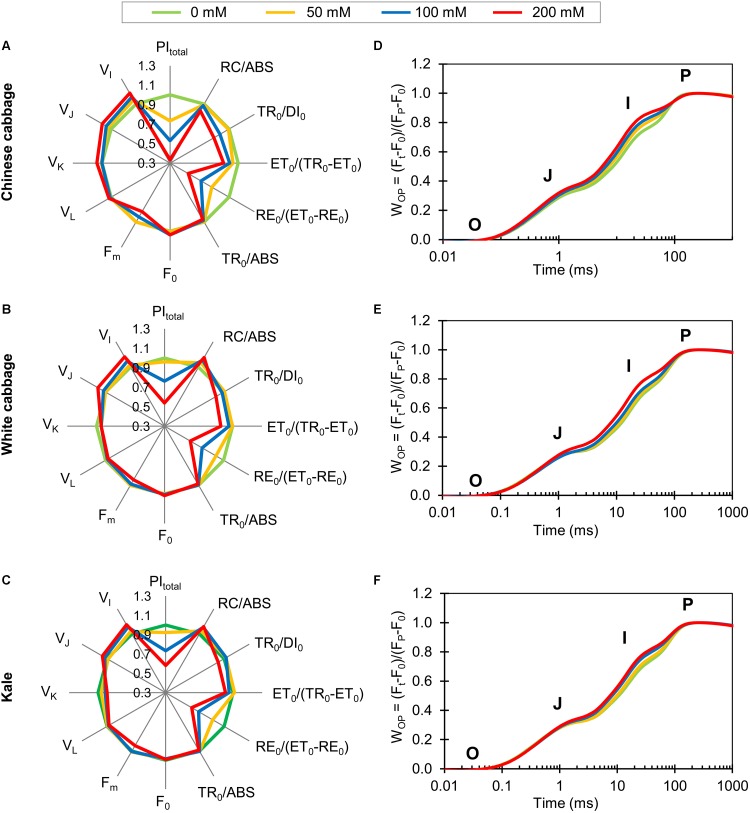
Spider plots and OJIP transients obtained from photosynthetic measurements of Chinese cabbage **(A,D)**, white cabbage **(B,E)**, and kale **(C,F)** exposed to NaCl at 50, 100, and 200 mM. The spider plots (panels **A–C**) show normalized values of selected parameters characterizing PSII functionality. All values shown are percentages of values obtained for control plants, enabling comparison of the variables measured on different scales. OJIP transients **(D–F)** are normalized between O and P steps: W_OP_ = (F_t_–F_0_)/(F_P_–F_0_). Each curve represents average kinetics of 18 replicates. The O, J, I, and P steps are marked in the plots. Raw data shown in spider plots are presented in Supplementary Table S3.

To acquire information about the selected brassicas’ photosynthetic performance, we measured fluorescence transients of hydroponically grown plants exposed to 50, 100, or 200 mM NaCl (Figure 2D–F). Normalized OJIP transients, indicating differences between control and stressed plants in PSII and PSI functionality, demonstrated that the fluorescence intensity at defined transient steps successively increased with increases in NaCl concentration in all three brassicas. Spider plots presenting normalized values of NaCl-treated plants relative to the controls are shown in Figure 2A–C. The plots show clear between-taxa differences in total photosynthetic performance index, PI_total_, a sensitive parameter reflecting the functional activity of PSII, PSI, and intersystem electron transport chain. The PI_total_ of Chinese cabbage decreased 26.6, 47, and 66.6% after exposure to 50, 100, and 200 NaCl, respectively, while decreases were only detected after exposure to 100 and 200 mM NaCl in white cabbage (23.8 and 46.2%, respectively) and kale (26.7 and 41.9%, respectively). The density of reaction centers (RCs) per unit chlorophyll *a*, RC/ABS, in Chinese cabbage decreased 8% at the highest NaCl concentration. In contrast, 100 and 200 mM NaCl induced significant increases in RC/ABS in white cabbage (8.4 and 11.5%, respectively) and kale (8.1 and 8.9%, respectively). Flux ratio trapping per dissipation, TR_0_/DI_0_, and electron transport further than Q_A_, ET_0_/(TR_0_-ET_0_), significantly declined in Chinese cabbage exposed to 100 and 200 mM NaCl (by 8.8 and 14.9%, respectively). In white cabbage and kale, these parameters decreased significantly only at the highest NaCl concentration, relative to controls (by 12.2 and 7.9%, respectively).

The probability of end electron acceptor reduction, RE_0_/ (ET_0_-RE_0_), decreased in Chinese cabbage by 20.6, 33.3, and 48.6% after exposure to 50, 100 and 200 NaCl, respectively. However, this parameter decreased only after exposure to 100 and 200 mM NaCl, relative to controls, in white cabbage (by 25.5 and 39.5%, respectively) and kale (by 30.5 and 38.9%, respectively).

Fluorescence intensity at 50 s (F_0_) was not affected in any cultivar by any of the salt treatments. The maximum fluorescence intensity (F_m_) was significantly decreased by exposure to 100 and 200 mM NaCl in Chinese cabbage (by 5.9 and 12.4%, respectively), while in white cabbage and kale it only significantly decreased at 200 mM (by 6.4 and 6.5%, respectively) relative to controls. Similarly, the maximum quantum yield of primary photochemistry, TR_0_/ABS, significantly decreased in Chinese cabbage at 100 and 200 mM NaCl relative to controls (by 1.7 and 3.1%, respectively), while in white cabbage and kale it decreased only at the highest NaCl concentration (by 1.5 and 1.2%, respectively).

Variable fluorescence at 150 μs (V_L_) showed no significant changes in any cultivar after exposure to salt at any concentrations relative to controls. Variable fluorescence at 300 μs (V_K_) significantly increased (by 5.3%) at the highest NaCl concentration in Chinese cabbage, while 100 and 200 mM NaCl caused significant reductions (7.2 and 9.2%, respectively) of this parameter in kale. There were significant increases in variable fluorescence at the J step (V_J_) in Chinese cabbage at 100 and 200 mM NaCl (5.9 and 10.7%, respectively), but only at 200 mM in white cabbage and kale (9.1 and 5.8%, respectively). Similarly, variable fluorescence at 30 s (V_I_) increased after exposure to 50, 100, and 200 mM NaCl in Chinese cabbage (by 5.0, 8.4, and 13.0%, respectively), but only after exposure to 100 and 200 mM NaCl in white cabbage (by 7.0 and 12.2%, respectively) and kale (by 8.2 and 11.1%, respectively).

**FIGURE 3:**
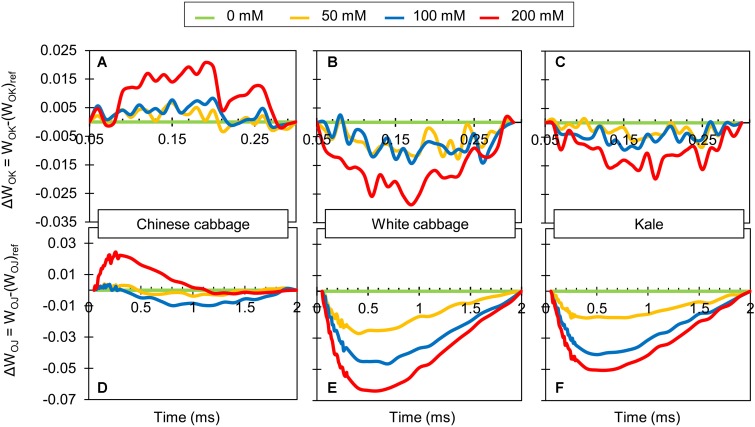
Chlorophyll *a* fluorescence transient curves obtained for leaves of Chinese cabbage **(A,D)**, white cabbage **(B,E)**, and kale **(C,F)** exposed to NaCl at 50, 100, and 200 mM. Each curve represents average kinetics of 18 replicates. Average fluorescence data were normalized between O and K steps (L-band; **A–C**) and plotted as difference kinetics ΔW_OK_ = W_OK_–(W_OK_)_ref_ in the 0.05–3 ms time range. Fluorescence data normalized between O and J steps (K-band; **D–F**) were plotted as difference kinetics ΔW_OJ_ = W_OJ_–(W_OJ_)_ref_ in the 0.05–2 ms time range. (W_OK_)_ref_ and (W_OJ_)_ref_ reference values were obtained from measurements of control plants for each species.

There were clear differences in L-bands (Figure 3A–C) and K-bands (Figure 3D–F), obtained from the normalized O-K and O-J curves between Chinese cabbage and the two other *Brassica* crops. Bands for Chinese cabbage were positive and their amplitude was highest after exposure to 200 mM NaCl. In contrast, both bands for white cabbage and kale were negative after exposure to NaCl at all concentrations, but again their amplitude was highest at 200 mM NaCl.

### Effects of Salinity on Biochemical and Physiological Stress Parameters

**FIGURE 4:**
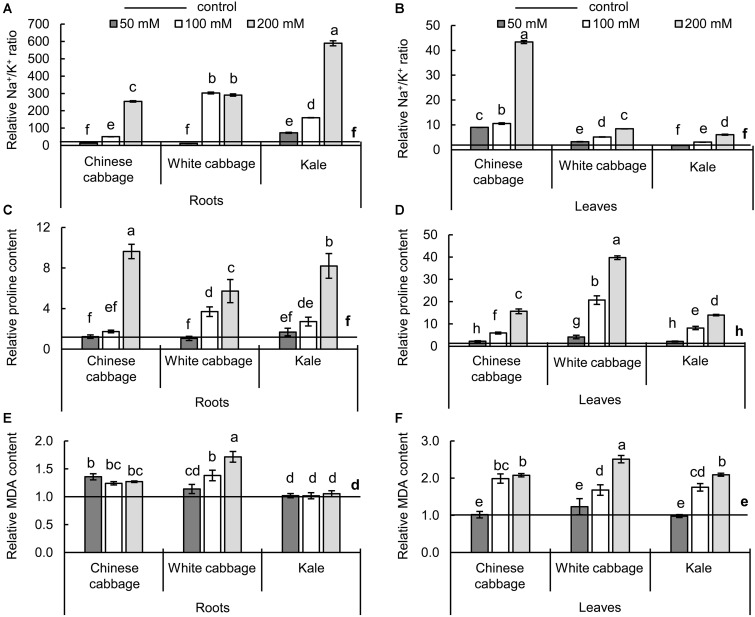
Na^+^/K^+^ ratios **(A,B)**, and contents of proline **(C,D)**, and malondialdehyde (MDA) **(E,F)** of the three *Brassica* crops in roots (left panels) and leaves (right panels) after exposure to 50, 100, and 200 mM NaCl, relative to NaCl-free controls. Normalized values of salt treatments presented as means ± SD, *n* = 4. Points labeled with different letters differ significantly at *p* < 0.05. Raw data are presented in Supplementary Table S2.

To evaluate effects of the treatments to major known biochemical salinity stress markers, the Na^+^/K^+^ ratio, and levels of proline and MDA (Figure 4) were measured in roots and leaves of the selected *Brassica* crops.

Maintenance of the potassium to sodium ionic ratio and accumulation of proline are salinity defense strategies. The increase of sodium ions and decrease of potassium ions (Supplementary Table S2) resulted in higher Na^+^/K^+^ ratio upon salinity (Figure 4A,B). The Na^+^/K^+^ ratio was affected more strongly in roots than in leaves, and exposure to the highest salt concentration (200 mM) resulted in 254-, 291-, and 589-fold changes in Chinese cabbage, white cabbage, and kale roots, respectively, relative to controls (Figure 4A). The highest difference in Na^+^/K^+^ ratio in leaves between control and salt treatments was 43-fold, in Chinese cabbage after exposure to 200 mM NaCl (Figure 4B), which induced ca. 10-fold increases in the Na^+^/K^+^ ratios of the other two brassicas’ leaves. The osmotic effect of NaCl was evaluated by monitoring changes in levels of proline (Figure 4C,D), which successively increased after exposure to 100 and 200 mM NaCl in both roots and leaves of the three crops. We detected 9.6-, 5.7-, and 8.2-fold increases in proline levels in roots of Chinese cabbage, white cabbage, and kale, respectively, after exposure to 200 mM NaCl, relative to controls (Figure 4C), and much higher increases in their leaves (15.6-, 39.7-, and 13.9-fold, respectively) (Figure 4D). To evaluate the oxidative effects of the treatments, MDA contents were measured (Figure 4E,F). The treatments did not significantly affect the MDA content in kale roots but significantly affected it in Chinese cabbage roots at all salt concentrations and white cabbage roots at higher salt concentrations (100 and 200 mM NaCl) (Figure 4E). Moreover, the two highest salt concentrations (100 and 200 mM) increased MDA levels in leaves of all three brassicas relative to corresponding controls (Figure 4F).

### Effects of Salinity on Stress Hormones

**FIGURE 5:**
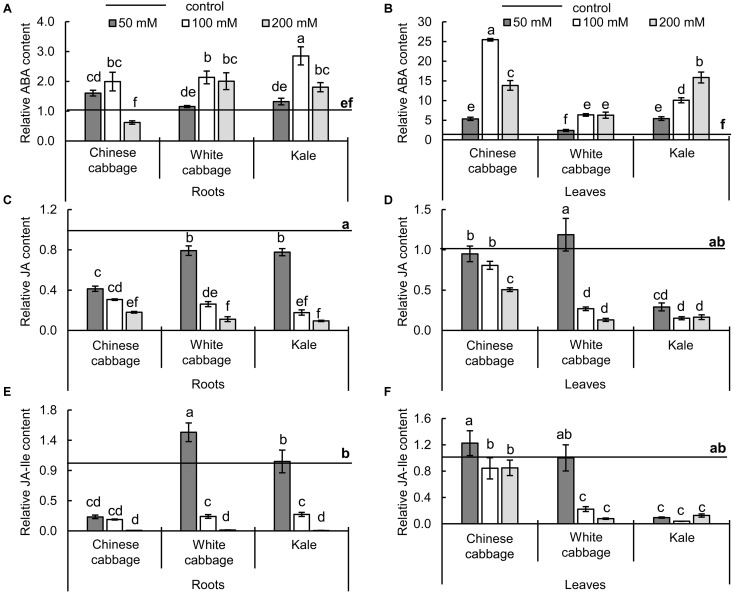
Levels of stress-related hormones – abscisic acid (ABA, **A,B**), jasmonic acid (JA, **C,D**), and jasmonoyl-L-isoleucine (JA-Ile, **E,F**) – in roots (left panels) and leaves (right panels) of Chinese cabbage, white cabbage, and kale after 24 h exposure to 50, 100, and 200 mM NaCl, relative to corresponding controls. Normalized values presented as means ± SD, *n* = 4. Points labeled with different letters differ significantly at *p* < 0.05. Raw data are presented in Supplementary Table S4.

Changes in stress-related hormones (ABA, JA, and JA-Ile) induced by the treatments are shown in Figure 5. Exposure to 100 and 200 mM NaCl caused significant increases in ABA levels in roots and leaves of all three brassicas (Figure 5A,B). The strongest increase in roots (2.85-fold relative to controls) was induced by exposure to 100 mM NaCl in roots of kale. However, the highest salinity (200 mM NaCl) decreased ABA levels in roots of Chinese cabbage (to 62% of control levels), although it increased them (approximately twofolds relative to controls) in white cabbage and kale roots. In leaves, the highest increase in ABA levels (25.5-fold) was also induced by 100 mM NaCl in Chinese cabbage (Figure 5B). The 200 mM NaCl treatment resulted in a smaller increase in ABA levels in Chinese cabbage, but similar and further increases in levels relative to those induced by 100 mM NaCl in leaves of white cabbage and kale, respectively. Levels of JAs (JA and JA-Ile) in roots and leaves (Figure 5C–F) showed similar responses to the salt stress treatments in all three crops. In mild stress conditions (50 mM NaCl) JA and JA-Ile (Figure 5D,F) remained at control levels in leaves of Chinese and white cabbage while levels of both hormones were significantly lower in kale. In root tissue, reductions in JA and JA-Ile contents (Figure 5C,E) were more pronounced in Chinese cabbage than in the other crops at 50 mM NaCl. Higher salinity resulted in significant decreases in levels of both hormones in roots and leaves of all three brassicas.

### Effects of Salinity on Brassinosteroids

**FIGURE 6:**
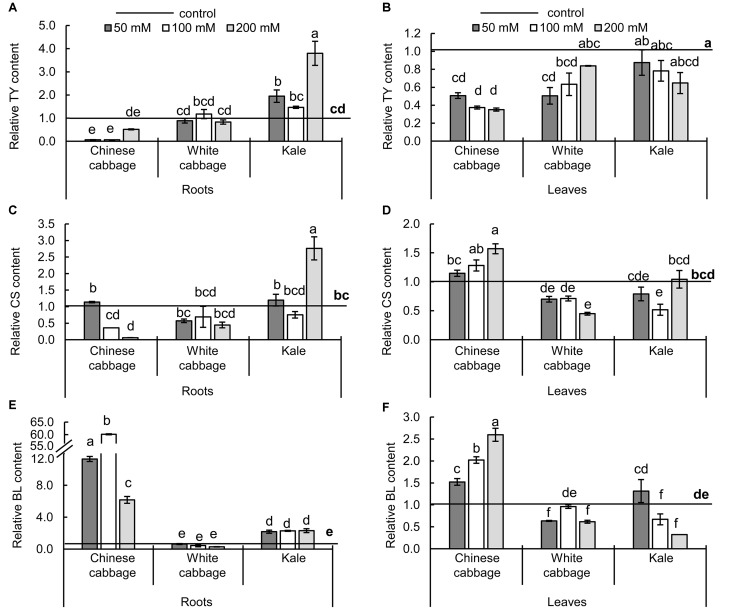
Levels of brassinosteroids – typhasterol (TY, **A,B**), castasterone (CS, **C,D**), and brassinolide (BL, **E,F**) – in roots (left panels) and leaves (right panels) of Chinese cabbage, white cabbage, and kale after 24 h exposure to 50, 100, and 200 mM NaCl, relative to controls. Normalized values presented as means ± SD, *n* = 3. Points labeled with different letters differ significantly at *p* < 0.05. Raw data are presented in Supplementary Table S5.

Changes in levels of BRs in the brassicas induced by the treatments are presented in Figure 6. In both roots and leaves, only three BRs (TY, CS, and BL) were detected. Reductions in levels of the precursor TY in roots and leaves (Figure 6A,B) were accompanied by increases in BL (Figure 6E,F) in both organs of Chinese cabbage exposed to NaCl at all concentrations, relative to controls. In contrast, TY, CS, and BL levels were moderately reduced in leaves and not affected in roots of white cabbage. The three stress treatments induced significant increases in levels of TY, CS, and BL in roots of kale (except in CS levels at 100 mM NaCl), but either no significant changes or reductions in kale leaves.

### Principal Component Analysis (PCA)

**FIGURE 7:**
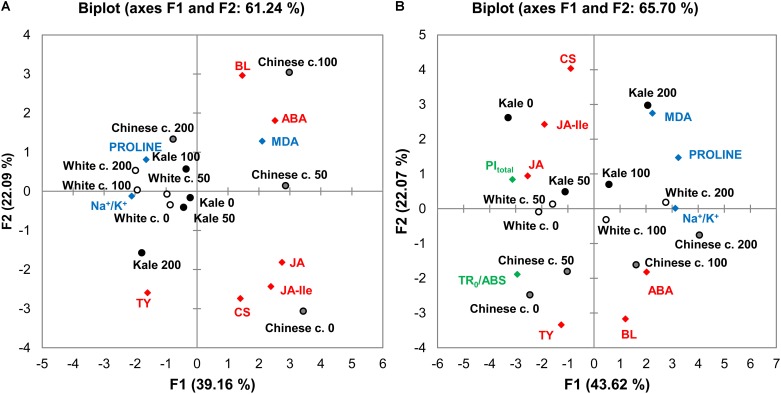
Results of two-dimensional principal component analysis (2D-PCA) of photosynthetic parameters, biochemical markers and phytohormone levels in roots **(A)** and leaves **(B)** of Chinese cabbage (Chinese c.), white cabbage (White c.), and kale (Kale) in roots **(A)** and leaves **(B)** after 24 h exposure to 0 (control), 50, 100, and 200 mM NaCl. Green, blue, and red symbols indicate positions in the score plots of the photosynthetic markers (Figure 2), biochemical parameters (Figure 4), and phytohormones (Figure 5, 6), respectively. Round symbols indicate positions of the species’ positions following each of the treatments. The correlation matrix used, eigenvalues, factor loadings and factor scores are given in Supplementary Tables S6–S15.

To investigate correlations among the brassicas in terms of the measured stress-related parameters, the data were subjected to PCA based on a matrix of Pearson correlation coefficients (*p* < 0.05). Average values of measured traits were standardized prior to analysis by autoscaling. The resulting correlation matrix, eigenvalues, factor loadings and factor scores are presented in Supplementary Tables S6–S20. Positions of the brassicas and relations among the measured parameters under the salinity treatments, in roots and leaves are shown in PCA biplots in Figure 7A,B, respectively. The first two Principal Components, F1 and F2, explained 61.24 and 65.70% of the cumulative variability of measured traits in the roots and leaves, respectively. Detailed PCA of correlations of photosynthetic parameters with the cultivars and treatments is presented in Supplementary Figure S2 in the Supplementary File and Supplementary Tables S16–S20. The first two Principal Components explained 79.85% of the cumulative variability of photosynthetic traits in leaves.

The separation of the cultivars, particularly Chinese cabbage, in the root biplot (Figure 7A) clearly shows that their salinity responses differ. Kale and white cabbage treatments were grouped close to their controls in the left quadrants, while Chinese cabbage treatments were positioned far away from the corresponding control. The PCA plot also shows relations of the measured parameters in the brassicas’ responses (factor loadings are presented in Supplementary Tables S8, S13 for roots and leaves, respectively). The phytohormones BL and ABA, and stress marker MDA are positioned close to 50 and 100 mM NaCl-treated Chinese cabbage roots, while proline is grouped together with 100 and 200 mM NaCl-treated white cabbage roots and Chinese cabbage at 200 mM NaCl, indicating that they are the main response parameters to these treatments. Parameters positioned in the same quadrant as kale roots and white cabbage under control and 50 mM NaCl treatments are TY and Na^+^/K^+^ ratio.

In the leaf biplot (Figure 7B), cultivars exposed to the control and mild stress (50 mM NaCl) treatments are grouped together on the left side of the plot, and the severe salinity treatments shifted them to the right. Like the pattern in the root biplot, Chinese cabbage leaves were positioned far from kale leaves, and white cabbage leaves exposed to the salinity treatments were in intermediate positions, indicating differences in their salinity responses and hence sensitivity to salt stress. The parameters positioned closest to Chinese cabbage exposed to severe salinity are the phytohormones ABA and BL, together with Na^+^/K^+^ ratio, while proline, and MDA are positioned closer to the other two *Brassica* varieties.

There were also significant correlations between phytohormones, biochemical, and selected photosynthetic parameters (PI_total_ and TR_0_/ABS) that reflect the overall efficiency of photosynthesis in leaves (Supplementary Table S11). These photosynthetic parameters were negatively correlated with the Na^+^/K^+^ ratio, proline and MDA, as well as ABA.

Based on photosynthetic parameters, *B. oleracea* cultivars (controls and salinity-treated) grouped close together and far from Chinese cabbage (Supplementary Figure S2 in the Supplementary File). PI_total_ was positively correlated with its constituents: TR_0_/DI_0_, ET_0_/(TR_0_-ET_0_), RE_0_/(ET_0_-RE_0_), and TR_0_/ABS, but negatively correlated with RC/ABS. It was also positively correlated with F_m_ but negatively correlated with V_J_ and V_I_. PI_total_ and its constituents were grouped close to control and mild salinity-treated *B. oleracea* varieties (white cabbage and kale), while the V_J_, V_L_, and V_I_ parameters were positioned closer to Chinese cabbage.

## Discussion

Soil salinization is a major agricultural problem, which impairs crops’ growth, yields, and quality. Soils with electrical conductivity (EC) of >4 dS/m are characterized as saline. However, under some environmental conditions, soil EC values can exceed 20 dS/m (corresponding to 200 mM NaCl) (Shannon and Grieve, 1999; Zhang et al., 2014). Thus, there are urgent needs to characterize crops’ salinity responses and the mechanisms involved in salinity tolerance. To aid such efforts we have examined, in detailed responses of selected brassicas to short-term salinity: Chinese cabbage (*B. rapa*), white cabbage (*B. oleracea* var. *capitata*) and kale (*B. oleracea* var. *acephala*). All three brassicas were grown in a hydroponic system until they reached the four fully developed leaf-stage, at which they are frequently transferred into fields in conventional cultivation systems and thus exposed to different environmental conditions. The selected salt concentrations (50, 100, and 200 mM NaCl) correspond to concentrations in naturally occurring saline soils. Diverse physiological, biochemical, and hormonal parameters were measured to gain insights into the brassicas’ responses to short-term salinity and correlations between their responses and tolerance.

### Salinity Tolerance and Photosynthetic Performance of the Selected Brassicas

An earlier study on *Brassica* crops identified root-growth inhibition as a significant biomarker of salt sensitivity(Zhang et al., 2014). Thus, we used a simple root-growth bioassay to characterize the selected cultivars’ salt sensitivity and found that Chinese cabbage was the most sensitive, while kale and white cabbage were more tolerant under the applied experimental conditions. These results are consistent with previous findings that cabbages (including *B. oleracea* var. *capitata*) have a salinity threshold of 180 mM NaCl, kales (such as *B. oleracea* var. *acephala*) can grow after exposure to 230–550 mM NaCl following re-watering (Shannon and Grieve, 1999), while many *B. rapa* sub-species have been described as salt-sensitive (Jan et al., 2016). As a component of salinity stress is osmotic stress, the results are also consistent with our recently published finding of a corresponding pattern of tolerance to drought conditions (Pavlović et al., 2018b).

Photosynthesis, and hence crops’ growth and productivity can be inhibited by salinity, to degrees that depend on the crops’ tolerance. Therefore, we tested the influence of salinity on the three brassicas’ photosynthetic performance by measuring their fast chlorophyll *a* fluorescence kinetics and determining several photosynthetic parameters. It is well known that OJIP kinetics, which reflects the redox state of Q_A_ and Q_B_, are extremely sensitive to salt stress (Mehta et al., 2010; Dabrowski et al., 2016; Duarte et al., 2017). The O-J phase is a light-dependent phase of OJIP kinetics that reflects reduction of the acceptor side of PSII (Schansker et al., 2014) and provides information on antenna size and connectivity between PSII RCs. Therefore, the significantly higher increase in variable fluorescence at the J step (V_J_) observed in Chinese cabbage in response to salinity stress than in the other two varieties can be attributed to a stronger decrease in re-oxidation of Q_A_. Between the O and J steps, two additional steps can be distinguished, designated the L- and K-bands (Yusuf et al., 2010). Positive L-bands obtained for salt-stressed Chinese cabbage, relative to controls (Figure 3A), indicate that its connectivity and system stability were impaired more than in the other two brassicas, for which negative L-bands were obtained. In addition, positive K-bands were obtained for salt-stressed Chinese cabbage (Figure 3D), presumably due to impaired electron flow between OEC and the acceptor side of the RC, while negative K-bands were obtained for salt-stressed white cabbage and kale (Yusuf et al., 2010; Chen et al., 2016). Negative K-bands have been recognized as signs of tolerance to stress conditions (Krüger et al., 2014; Żurek et al., 2014; Begović et al., 2016), indicating that PSII antennae are functionally intact (Venkatesh et al., 2012). Further, the J-I phase reflects a partial reduction of the intersystem electron carriers, while the I-P phase reflects reduction of the acceptor side of PSI (Yusuf et al., 2010; Schansker et al., 2014). The increase in V_I_ induced by all salinity treatments in Chinese cabbage (Figure 2A) is attributable to faster accumulation of reduced Q_A_ and Q_B_-non-reducing PSII centers that cannot transfer electrons further along the electron transport chain (Tomek et al., 2001), suggesting that even the mildest salt stress partially inactivated its PSII centers. In contrast, indications of restricted electron transport between Q_A_ and Q_B_ (increases in V_I_) in white cabbage and kale were only detected following exposure to higher NaCl concentrations (Figure 2B,C). Furthermore, salinity exposure decreased the photosynthetic system’s ability to convert excitation energy to electron transport beyond Q_A._ This is consistent with a recent demonstration that increases in V_I_ are due to failure of PSI to oxidize reduced plastoquinone (Goltsev et al., 2016).

Exposure to salt stress did not induce changes in the F_0_ parameter in any *Brassica*, while the F_m_ parameter decreased more strongly in Chinese cabbage than in the others (Figure 2) suggesting inhibition of electron flow through PSII. Reductions in the maximum yield of PSII primary photochemistry, TR_0_/ABS (Figure 3A–C) was obtained in all three *Brassica* cultivars, indicating impairment of PSII photochemical efficiency. This is supported by a recent report of similar changes in F_0_, F_m_ and TR_0_/ABS parameters in two perennial ryegrasses exposed to salt stress (Dabrowski et al., 2016).

The most sensitive parameter of the JIP-test is the total photosynthetic performance index, PI_total_ (Figure 2A–C), which indicates the overall functional activity of both photosystems, PSII and PSI, as well as the intersystem electron transport chain (Yusuf et al., 2010; Krüger et al., 2014; Dabrowski et al., 2016; Goltsev et al., 2016; Kalaji et al., 2017). This multiparametric expression reflects the overall efficiency of light energy absorption (RC/ABS), quantum yield of excitation energy trapping (TR_0_/DI_0_), probability of a trapped exciton moving an electron further along the electron transport chain than Q_A_ [ET_0_/(TR_0_-ET_0_)] and the probability of PSI reducing its end acceptors [RE_0_/(ET_0_-RE_0_)] (Yusuf et al., 2010; Krüger et al., 2014). PI_total_ declined in all three *Brassica* crops exposed to salinity (Figure 2A–C), but most strongly in Chinese cabbage. The most sensitive PI_total_ component to the salinity treatments was the RE_0_/( ET_0_-RE_0_) parameter, reflecting the efficiency of processes involving PSI and its ability to reduce its end acceptors. In addition, reductions in TR_0_/DI_0_ and ET_0_/(TR_0_-ET_0_) were observed in all three brassicas at the highest NaCl concentration, and in Chinese cabbage reductions also occurred at 100 mM NaCl. This suggests that the higher NaCl concentrations caused structural damage to thylakoids, thereby reducing trapping of excitation energy and its conversion to electron transport. Similarly, Dabrowski et al. (2016) reported that diminution of PSII activity and impairment of PSI function significantly decreased the total performance index in ryegrass, and Oukarroum et al. (2015) detected salt-induced inhibition of both PSII and PSI electron transport activities in duckweed. In addition, Mehta et al. (2010) suggested that salt stress decreased efficient electron transport further than the primary acceptor, and it can significantly decrease values of all parameters related to electron transport in tomato leaves (Zushi and Matsuzoe, 2017).

### Salt Stress-Related Biochemical Parameters in the Selected Brassicas

An important component of plants’ salt tolerance is the maintenance of an appropriate cytosolic Na^+^/K^+^ ratio, and the tolerance of *Brassica* genotypes reportedly correlates inversely with Na^+^/K^+^ ratios in their roots, steam, and leaves when exposed to salinity. Salt tolerance is also reportedly correlated with transcription levels of genes involved in ionic homeostasis, including components of the Salt Overly Sensitive (SOS) response and vacuolar NHX1 Na^+^/H^+^ antiporter responsible for pumping Na^+^ into vacuoles from the cytoplasm (Chakraborty et al., 2012). Furthermore, efficient compartmentalization of Na^+^ into vacuoles and regulation of Na^+^ transport from roots to shoots is crucial for plant survival since Na^+^ is more toxic to leaves (Almeida et al., 2017; Assaha et al., 2017). These reports are consistent with our findings that white cabbage and kale had lower Na^+^/K^+^ ratios in their leaves than the salt-sensitive Chinese cabbage, mainly due to increases in Na^+^ contents. Observations of lower Na^+^/K^+^ ratios in leaves and higher ratios in roots of white cabbage and kale suggest that control of Na^+^ transport from roots to leaves is more efficient in these cultivars.

In addition to ionic homeostasis, osmoregulation of cell turgor is another important physiological process under salt stress. We observed a positive correlation between the Na^+^/K^+^ ratio and proline content. Osmoregulatory accumulation of proline under saline conditions is reportedly a common response of glycophytes, including many brassicas (Kumar et al., 2009; Mittal et al., 2012; Jan et al., 2016). Higher concentrations of proline accumulated in leaves than in roots, while the Na^+^ levels were much higher in roots of the selected brassicas. Similar salinity responses have been recorded in tomato plants, where proline accumulation positively correlated with expression of the *P5CS* (proline biosynthesis) gene and accumulation of Na^+^ in leaves, but not in roots (Almeida et al., 2014). The cited authors concluded that there is no link between proline accumulation and a plant tolerance index, suggesting that proline “is not, *per se*, the driving force of tolerance.” Similarly, we found that kale leaves accumulated less proline than the other two, more sensitive brassicas. Some studies suggest that proline contributes to redox homeostasis, and increases in its biosynthesis help maintain functionality of the photosynthetic apparatus and induce increases in activities of antioxidant enzymes (Puniran-Hartley et al., 2014; Surender Reddy et al., 2015). However, Pearson’s coefficients we obtained show a negative correlation between the PI_total_ photosynthetic index and proline, suggesting that proline does not play a major protective role in photosynthesis in the selected *Brassica* cultivars under our experimental conditions. Furthermore, increases in MDA levels (a marker of lipid peroxidation) suggest that salt stress increases ROS production in leaves of all three brassicas more than in roots. The pro-oxidant character of high endogenous proline levels (Soshinkova et al., 2013) in leaves or insufficient antioxidant machinery could potentially explain our findings.

### Roles of Phytohormones in Salinity Responses of the Selected Brassicas

Early stress responses are mediated by complex crosstalk of phytohormones (including synergistic and antagonistic interactions), among which ABA is reportedly the main salinity messenger (Fahad et al., 2015). Links between ABA and salinity tolerance are widespread in the plant kingdom but highly dependent on genotype, developmental stage, and stress conditions. Increases in ABA in responses to salinity have been detected in sensitive genotypes of tomato (Duan et al., 2012), basil (Mancarella et al., 2016), and barley (Witzel et al., 2014). However, significant increases in ABA have also been recorded in early responses of a tolerant barley variety (Kamboj et al., 2015) and sustained responses of a tolerant tomato cultivar (Gharbi et al., 2017). According to Ellouzi et al. (2014), the halophytes *Cakile maritima* and *Thellungiella salsuginea* can accumulate ABA more efficiently than the glycophyte *A. thaliana* under extreme salinity (400 mM NaCl). These findings are consistent with our results. The moderately tolerant white cabbage and tolerant kale maintained or dose-dependently increased levels of ABA in their leaves in response to salt. In contrast, ABA levels were lower than control levels in roots of the salt-sensitive Chinese cabbage at 200 mM NaCl, and lower in its leaves at 200 mM than at 100 mM NaCl. Moreover, observed decreases in the photosynthetic parameter PI_total_, particularly in Chinese cabbage leaves, are consistent with control of transpiration in salinity responses through ABA-induced stomatal closure and associated reductions in photosynthetic rates (Ashraf and Harris, 2013).

Furthermore, the observed accumulation of proline following exposure to salinity can be partially explained by increased levels of ABA since proline biosynthesis is regulated by an ABA-dependent and an ABA-independent pathway (Strizhov et al., 1997; Verslues and Bray, 2006). Thus, the observed discrepancy between proline accumulation and changes in ABA levels implies that the selected brassicas respond to salinity through different osmoregulatory mechanisms, which are organ- and cultivar-specific. Moreover, ABA is involved in the SOS pathway and prevention of Na^+^ transport from roots to shoots through xylem (Zhu et al., 2017). Reduced ABA levels in Chinese cabbage roots, accompanied by a sharp increase in the Na^+^/K^+^ ratio in leaves at the highest salinity, show that Chinese cabbage has less ability to control Na^+^ leakage to shoots than the other two more tolerant cultivars.

In addition to ABA, JAs contribute to abiotic stress responses, although their roles in responses to biotic stress have received the most attention. There are also conflicting indications of their roles in salinity responses. Increases in JA levels following exposure to salinity, with associated increases in tolerance, have been recorded in several species, including tomato, iris, and rice (Wang et al., 2001; Pedranzani et al., 2003; Tani et al., 2008). However, impaired biosynthesis of the JA precursor OPDA reportedly enhances the stress tolerance of *Arabidopsis* *aoc* mutants by increasing ROS scavenging activity and delaying senescence. These mutants also reportedly accumulate less Na^+^ in shoots than wild type controls when exposed to salinity, while their roots’ ion contents are not affected (Hazman et al., 2015). Similarly, we found that in leaves of more tolerant cultivars exposed to severe salinity, contents of JA-Ile (the most bioactive jasmonate) were lower than in corresponding controls, while JA-Ile levels in leaves of salt-sensitive Chinese cabbage were not influenced by salinity. Furthermore, Kurotani et al. (2015) found that the bioactive JAs negatively affect rice viability under salt stress in experiments with a transgenic mutant. Enhanced expression of CYP94C2b, which contributes to the conversion of bioactive JA-Ile to the inactive forms 12OH-JA-Ile and 12COOH-JA-Ile, did not affect natural senescence but delayed salt-induced senescence in an overexpressing rice mutant. The repression of JA-signaling is maintained at low levels of JA-Ile, while increases in its levels trigger JA-signaling (Wasternack and Strnad, 2016). Therefore, the considerably lower amounts of JA and JA-Ile we observed in leaves of white cabbage and kale may potentially contribute to a postponement of JA-mediated senescence in saline conditions.

The role of BRs in salt stress has been explored through monitoring effects of exogenous application of active BRs, which reportedly increases plants’ tolerance, mainly through induction of H_2_O_2_ signaling and antioxidant machinery (Sharma et al., 2017). BRs can also mitigate some negative effects of salt stress on photosynthesis (Siddiqui et al., 2018). However, little is known about the roles of endogenous BRs in salinity stress responses. The *Arabidopsis* BR signaling impaired mutants BR-deficient *det2-1* and BR-insensitive *bin2-1* reportedly have higher sensitivity to salt stress during seed germination and seedling growth than wild type counterparts and have reduced levels of proline and transcripts of salt- and ABA-induced genes (Zeng et al., 2010). However, improved drought tolerance and delays in senescence during drought have been observed in transgenic creeping bentgrass (*Agrostis stolonifera* L.) with reduced levels of BRs (Han et al., 2017). Correlations between reduced levels of BL and improvements in drought tolerance have also been recorded in *Arabidopsis* plants (Northey et al., 2016). Moreover, we recently found that more BL accumulated in drought-sensitive Chinese cabbage than in the more tolerant kale and white cabbage in drought conditions (Pavlović et al., 2018b). Furthermore, a positive correlation between ABA and BL was noted. It is known that stomata closure can be mediated by BR and stress hormone (ABA, ethylene) crosstalk (Ha et al., 2016; Jiroutova et al., 2018), which may lead to reduced photosynthetic rates under salinity stress (Ashraf and Harris, 2013). Since photosynthesis was most strongly affected in Chinese cabbage, changes in BR levels could be linked to the osmotic component of salt stress. Accumulation of BRs was also observed in roots; kale accumulated three times more CS at the highest salinity while BL levels in Chinese cabbage were increased up to 60-fold, depending on the treatment. The high accumulation of BL in this salt-sensitive cultivar correlates with its stronger inhibition of root growth observed in the root-growth bioassay. In addition, experiments with *Arabidopsis* *bes1-D* mutants, which have enhanced BR signaling, and treatment of wild type plants with high levels of BL, have revealed that BRs promote overall root growth inhibition (González-García et al., 2011). Maintenance of optimal levels of active BRs in roots may contribute to tolerance mechanisms since it has been proposed that BR signaling is temporarily inhibited in early phases of root growth response to salinity (Geng et al., 2013).

## Conclusion

Based on our data, Chinese cabbage is the most sensitive, white cabbage moderately while kale the most tolerant to salinity stress among selected brassicas. Reductions in photosynthetic efficiency (PI_total_) were observed in all three brassicas exposed to higher salt concentrations, indicating that salt stress reduced the capacity of the photosynthetic system for efficient energy conversion, particularly in Chinese cabbage. This is in agreement with biomass reduction upon prolonged salinity stress (7 days) which was the most prominent in Chinese cabbage, then in white cabbage and finally kale. ABA levels were enhanced in all three brassicas under salt stress which is consistent with control of transpiration in salinity responses through ABA-induced stomatal closure and associated reductions in photosynthetic rates. It was shown that more tolerant varieties were able to sustain or even increase their ABA levels, while those of the sensitive Chinese cabbage declined under the most severe salt conditions. Better tolerance of kale was accompanying with better ability to control Na^+^ leakage from roots to shoots; Na^+^/K^+^ ratio was high in root and low in leaves with low MDA level at the same time in comparison to more sensitive brassicas. Furthermore, the considerably lower amounts of JA and JA-Ile measured in leaves of more tolerant white cabbage and kale may potentially contribute to the postponement of JA-mediated senescence in saline conditions. Finally, enhanced salinity tolerance in selected brassicas is accompanying with a higher level of TY (precursor of active BRs) and lower level of active BL. The high accumulation of BL in salt-sensitive cultivar correlates with its stronger root growth inhibition and reduced photosynthetic rates under salinity stress.

To our knowledge, this is the first report on comparative research on some *Brassica* crops to gain insights into correlations between initial salinity tolerance and diverse physiological, biochemical and hormonal parameters in Brassicaceae. However, long-term exposure of *Brassica* crops to salinity stress need to be investigated and compared to these initial salt responses. Further molecular-level research is needed to establish more precise conclusions and global understanding of salinity tolerance in brassicas.

## Author Contributions

IP and BS-S designed the research. IP performed the salinity stress experiments, analyzed the levels of stress hormones, conducted the statistical and principle component analyses, and designed the figures. BS-S performed the root-growth bioassays and biomass production experiments. JO and DT performed the brassinosteroid instrumental and data analyses. ON and MS supervised all other hormonal measurements and data analysis. VVB and SRB performed the stress diagnostic experiments and data analysis. HL performed the photosynthesis measurements. SM was responsible for analysis of photosynthesis data, statistics, and figure design. IP and SM drafted the manuscript. All authors discussed the results and implications, edited the manuscript, and approved the final manuscript.

## Conflict of Interest Statement

The authors declare that the research was conducted in the absence of any commercial or financial relationships that could be construed as a potential conflict of interest.

## References

[B1] Ahmad P. Rasool S. Gul A. Sheikh S. A. Akram N. A. Ashraf M. (2016). Jasmonates: multifunctional roles in stress tolerance. *Front. Plant Sci.* 7:813. 10.3389/fpls.2016.00813 27379115 PMC4908892

[B2] Almeida D. M. Margarida Oliveira M. Saibo N. J. M. (2017). Regulation of Na^+^ and K^+^ homeostasis in plants: towards improved salt stress tolerance in crop plants. *Genet. Mol. Biol.* 40 326–345. 10.1590/1678-4685-GMB-2016-0106 28350038 PMC5452131

[B3] Almeida P. Feron R. de Boer G. J. de Boer A. H. (2014). Role of Na^+^, K^+^, Cl^-^, proline and sucrose concentrations in determining salinity tolerance and their correlation with the expression of multiple genes in tomato. *AoB Plants* 6:plu039. 10.1093/aobpla/plu039 24996430 PMC4122256

[B4] Ashraf M. Harris P. J. C. (2013). Photosynthesis under stressful environments: an overview. *Photosynthetica* 51 163–190. 10.1111/plb.12014 23574304

[B5] Assaha D. V. M. Ueda A. Saneoka H. Al-Yahyai R. Yaish M. W. (2017). The role of Na^+^ and K^+^ transporters in salt stress adaptation in glycophytes. *Front. Physiol.* 8:509 10.3389/fphys.2017.00509 PMC5513949 28769821

[B6] Begović L. Mlinarić S. Dunić J. A. Katanić Z. Lončarić Z. Lepeduš H. (2016). Response of *Lemna minor* L. to short-term cobalt exposure: the effect on photosynthetic electron transport chain and induction of oxidative damage. *Aquat. Toxicol.* 175 117–126. 10.1016/j.aquatox.2016.03.009 27015565

[B7] Chakraborty K. Sairam R. K. Bhattacharya R. C. (2012). Differential expression of salt overly sensitive pathway genes determines salinity stress tolerance in *Brassica* genotypes. *Plant Physiol. Biochem.* 51 90–101. 10.1016/j.plaphy.2011.10.001 22153244

[B8] Chen S. Yang J. Zhang M. Strasser R. J. Qiang S. (2016). Classification and characteristics of heat tolerance in *Ageratina adenophora* populations using fast chlorophyll a fluorescence rise O-J-I-P. *Environ. Exp. Bot.* 122 126–140. 10.1016/j.envexpbot.2015.09.011

[B9] Dabrowski P. Baczewska A. H. Pawluśkiewicz B. Paunov M. Alexantrov V. Goltsev V. (2016). Prompt chlorophyll a fluorescence as a rapid tool for diagnostic changes in PSII structure inhibited by salt stress in *Perennial ryegrass*. *J. Photochem. Photobiol. B Biol.* 157 22–31. 10.1016/j.jphotobiol.2016.02.001 26878219

[B10] Duan H. Zhu Y. Qi D. Li W. Hua X. Liu Y. (2012). Comparative study on the expression of genes involved in carotenoid and ABA biosynthetic pathway in response to salt stress in tomato. *J. Integr. Agr.* 11 1093–1102. 10.1016/S2095-3119(12)60102-6

[B11] Duarte B. Cabrita M. T. Gameiro C. Matos A. R. Godinho R. Marques J. C. (2017). Disentangling the photochemical salinity tolerance in *Aster tripolium* L.: connecting biophysical traits with changes in fatty acid composition. *Plant Biol.* 19 239–248. 10.1111/plb.12517 27748562

[B12] Ellouzi H. Ben Hamed K. Hernández I. Cela J. Müller M. Magné C. (2014). A comparative study of the early osmotic, ionic, redox and hormonal signaling response in leaves and roots of two halophytes and a glycophyte to salinity. *Planta* 240 1299–1317. 10.1007/s00425-014-2154-7 25156490

[B13] Fahad S. Hussain S. Matloob A. Khan F. A. Khaliq A. Suad S. (2015). Phytohormones and plant responses to salinity stress: a review. *Plant Growth Regul.* 75 391–404. 10.1007/s10725-014-0013-y

[B14] Fiket Ž. Mikac N. Kniewald G. (2016). Mass fractions of forty-six major and trace elements, including rare earth elements, in sediment and soil reference materials used in environmental studies. *Geostand. Geoanal. Res.* 41 123–135. 10.1111/ggr.12129

[B15] Finkelstein R. (2013). Abscisic acid synthesis and response. *Arabidopsis Book* 11:e0166. 10.1199/tab.0166 24273463 PMC3833200

[B16] Floková K. Tarkowská D. Miersch O. Strnad M. Wasternack C. Novák O. (2014). UHPLC-MS/MS based target profiling of stress-induced phytohormones. *Phytochemistry* 105 147–157. 10.1016/j.phytochem.2014.05.015 24947339

[B17] Geng Y. Wu R. Wee C. W. Xie F. Wei X. Chan P. M. Y. (2013). A spatio-temporal understanding of growth regulation during the salt stress response in *Arabidopsis*. *Plant Cell* 25 2132–2154. 10.1105/tpc.113.112896 23898029 PMC3723617

[B18] Gharbi E. Martínez J. P. Benahmed H. Hichri I. Dobrev P. I. Motyka V. (2017). Phytohormone profiling in relation to osmotic adjustment in NaCl-treated plants of the halophyte tomato wild relative species *Solanum chilense* comparatively to the cultivated glycophyte *Solanum lycopersicum*. *Plant Sci.* 258 77–89. 10.1016/j.plantsci.2017.02.006 28330565

[B19] Goltsev V. Kalaji H. Paunov M. Baba W. Horaczek T. Mojski J. (2016). Variable chlorophyll fluorescence and its use for assessing physiological condition of plant photosynthetic apparatus. *Russ. J. Plant Physiol.* 63 869–893. 10.1134/S1021443716050058

[B20] González-García M. P. Vilarrasa-Blasi J. Zhiponova M. Divol F. Mora-Garcia S. Russinova E. (2011). Brassinosteroids control meristem size by promoting cell cycle progression in *Arabidopsis* roots. *Development* 138 849–859. 10.1242/dev.057331 21270057

[B21] Ha Y. Shang Y. Nam K. H. (2016). Brassinosteroids modulate ABA-induced stomatal closure in *Arabidopsis*. *J. Exp. Bot.* 67 6297–6308. 10.1093/jxb/erw385 27856707 PMC5181576

[B22] Han J. Y. Kim Y. S. Hwang O. J. Roh J. Ganguly K. Kim S. K. (2017). Overexpression of *Arabidopsis thaliana* brassinosteroid-related acyltransferase 1 gene induces brassinosteroid-deficient phenotypes in creeping bentgrass. *PLoS One* 12:e0187378. 10.1371/journal.pone.0187378 29084267 PMC5662239

[B23] Hazman M. Hause B. Eiche E. Nick P. Riemann M. (2015). Increased tolerance to salt stress in OPDA-deficient rice ALLENE OXIDE CYCLASE mutants is linked to an increased ROS-scavenging activity. *J. Exp. Bot.* 66 3339–3352. 10.1093/jxb/erv142 25873666 PMC4449546

[B24] Jajoo A. (2014). “Changes in photosystem II heterogeneity in response to high salt stress,” in *Contemporary Problems of Photosynthesis*, eds Allakhverdiev S. I. Rubin A. B. Shuvalov V. A. (Izhevsk-Moscow: Institute of Computer Science), 397–413.

[B25] Jan A. S. Shinwari Z. K. Rabbani M. A. (2016). Morpho-biochemical evaluation of *Brassica rapa* sub-species for salt tolerance. *Genetika* 48 323–338. 10.2298/GENSR1601323J

[B26] Jiroutova P. Okleskova J. Strnad M. (2018). Crosstalk between brassinosteroids and ethylene during plant growth and under abiotic stress conditions. *Int. J. Mol. Sci.* 19:E3283. 10.3390/ijms19103283 30360451 PMC6214044

[B27] Julkowska M. M. Testerink C. (2015). Tuning plant signaling and growth to survive salt. *Trend Plant Sci.* 20 586–594. 10.1016/j.tplants.2015.06.008 26205171

[B28] Kalaji H. M. Bosa K. Kościelniak J. Żuk-Gołaszewska K. (2011). Effects of salt stress on photosystem II efficiency and CO2 assimilation of two Syrian barley landraces. *Environ. Exp. Bot.* 73 64–72. 10.1016/j.envexpbot.2010.10.009

[B29] Kalaji H. M. Račková L. Paganová V. Swoczyna T. Rusinowski S. Sitko K. (2017). Can chlorophyll a fluorescence parameters be used as bio-indicators to distinguish between drought and salinity stress in *Tilia cordata* mill? *Environ. Exp. Bot.* 152 149–157. 10.1016/j.envexpbot.2017.11.001

[B30] Kamboj A. Ziemann M. Bhave M. (2015). Identification of salt-tolerant barley varieties by a consolidated physiological and molecular approach. *Acta Physiol. Plant.* 37:1716 10.1007/s11738-014-1716-4

[B31] Krüger G. H. J. De Villiers M. F. Strauss A. J. de Beer M. van Heerden P. D. R. Maldonado R. (2014). Inhibition of photosystem II activities in soybean (*Glycine max*) genotypes differing in chilling sensitivity. *S. Afr. J. Bot.* 95 85–96. 10.1016/j.sajb.2014.07.010

[B32] Kumar G. Purty R. S. Sharma M. P. Singla-Pareek S. L. Pareek A. (2009). Physiological responses among *Brassica* species under salinity stress show strong correlation with transcript abundance for SOS pathway-related genes. *J. Plant Physiol.* 166 507–520. 10.1016/j.jplph.2008.08.001 18799232

[B33] Kurotani K. I. Hayashi K. Hatanaka S. Toda Y. Ogawa D. Ichikawa H. (2015). Elevated levels of CYP94 family gene expression alleviate the jasmonate response and enhance salt tolerance in rice. *Plant Cell Physiol.* 56 779–789. 10.1093/pcp/pcv006 25637374

[B34] Liang W. Ma X. Wan P. Liu L. (2018). Plant salt-tolerance mechanism: a review. *Biochem. Biophys. Res. Commun.* 495 286–291. 10.1016/j.bbrc.2017.11.043 29128358

[B35] Mancarella S. Orsini F. Van Oosten M. J. Sanoubar R. Stanghellini C. Kondo S. (2016). Leaf sodium accumulation facilitates salt stress adaptation and preserves photosystem functionality in salt stressed *Ocimum basilicum*. *Environ. Exp. Bot.* 130 162–173. 10.1016/j.envexpbot.2016.06.004

[B36] Mehta P. Jajoo A. Mathur S. Bharti S. (2010). Chlorophyll a fluorescence study revealing effects of high salt stress on photosystem II in wheat leaves. *Plant Physiol. Biochem.* 48 16–20. 10.1016/j.plaphy.2009.10.006 19932973

[B37] Mittal S. Kumari N. Sharma V. (2012). Differential response of salt stress on *Brassica juncea*: photosynthetic performance, pigment, proline, D1 and antioxidant enzymes. *Plant Physiol. Biochem.* 54 17–26. 10.1016/j.plaphy.2012.02.003 22369937

[B38] Munns R. Gilliham M. (2015). Salinity tolerance of crops - what is the cost? *New Phytol.* 208 668–673. 10.1111/nph.13519 26108441

[B39] Munns R. James R. A. Gilliham M. Flowers T. J. Colmer T. D. (2016). Tissue tolerance: an essential but elusive trait for salt-tolerant crops. *Funct. Plant Biol.* 43 1103–1113. 10.1071/FP16187 32480530

[B40] Northey J. G. Liang S. Jamshed M. Deb S. Foo E. Reid J. B. (2016). Farnesylation mediates brassinosteroid biosynthesis to regulate abscisic acid responses. *Nat. Plants* 2:16114. 10.1038/nplants.2016.114 27455172

[B41] Oklestkova J. Tarkowská D. Eyer L. Elbert T. Marek A. Smržová Z. (2017). Immunoaffinity chromatography combined with tandem mass spectrometry: a new tool for the selective capture and analysis of brassinosteroid plant hormones. *Talanta* 170 432–440. 10.1016/j.talanta.2017.04.044 28501193

[B42] Oukarroum A. Bussotti F. Goltsev V. Kalaji H. M. (2015). Correlation between reactive oxygen species production and photochemistry of photosystems I and II in *Lemna gibba* L. plants under salt stress. *Environ. Exp. Bot.* 109 80–88. 10.1016/j.envexpbot.2014.08.005

[B43] Pavlović I. Pěnčík A. Novák O. Vujčić V. Radić Brkanac S. Lepeduš H. (2018a). Short-term salt stress in Brassica rapa seedlings causes alterations in auxin metabolism. *Plant Physiol. Biochem.* 125 74–84. 10.1016/j.plaphy.2018.01.026 29427890

[B44] Pavlović I. Petřik I. Tarkowská D. Lepeduš H. Vujčić Bok V. Radić Brkanac S. (2018b). Correlations between phytohormones and drought tolerance in selected Brassica crops: Chinese cabbage, white cabbage and kale. *Int. J. Mol. Sci.* 19:2866. 10.3390/ijms19102866 30241414 PMC6213169

[B45] Pedranzani H. Racagni G. Alemano S. Miersch O. Ramírez I. Peña-Cortés H. (2003). Salt tolerant tomato plants show increased levels of jasmonic acid. *Plant Growth Regul.* 4 149–158. 10.1080/15592324.2016.1146847 26906266 PMC5703232

[B46] Per T. S. Khan M. I. R. Anjum N. A. Masood A. Hussain S. J. Khan N. A. (2018). Jasmonates in plants under abiotic stresses: crosstalk with other phytohormones matters. *Environ. Exp. Bot.* 145 104–120. 10.1016/j.envexpbot.2017.11.004

[B47] Puniran-Hartley N. Hartley J. Shabala L. Shabala S. (2014). Salinity-induced accumulation of organic osmolytes in barley and wheat leaves correlates with increased oxidative stress tolerance: in planta evidence for cross-tolerance. *Plant Physiol. Biochem.* 83 32–39. 10.1016/j.plaphy.2014.07.005 25068241

[B48] Purty R. S. Kumar G. Singla-Pareek S. L. Pareek A. (2008). Towards salinity tolerance in Brassica: an overview. *Physiol. Mol. Biol. Plants* 14 39–49. 10.1007/s12298-008-0004-4 23572872 PMC3550665

[B49] Radić S. Cvjetko P. Glavaš K. Roje V. Pevalek-Kozlina B. Pavlica M. (2009). Oxidative stress and DNA damage in broad bean (*Vicia faba* L.) seedlings induced by thallium. *Environ. Toxicol. Chem.* 28 189–196. 10.1897/08-188.1 18717625

[B50] Raja V. Majeed U. Kang H. Andrabi K. I. John R. (2017). Abiotic stress: interplay between ROS, hormones and MAPKs. *Environ. Exp. Bot.* 137 142–157. 10.1016/j.envexpbot.2017.02.010

[B51] Riemann M. Dhakarey R. Hazman M. Miro B. Kohli A. Nick P. (2015). Exploring jasmonates in the hormonal network of drought and salinity. *Front. Plant Sci.* 6:1077. 10.3389/fpls.2015.01077 26648959 PMC4665137

[B52] Schansker G. Tóth S. Z. Holzwarth A. R. Garab G. (2014). Chlorophyll a fluorescence: beyond the limits of the QA model. *Photosyn. Res.* 120 43–58. 10.1007/s11120-013-9806-5 23456268

[B53] Shannon M. C. Grieve C. M. (1999). Tolerance of vegetable crops to salinity. *Sci. Hortic.* 78 5–38. 10.1016/S0304-4238(98)00189-7

[B54] Sharma I. Kaur N. Pati P. K. (2017). Brassinosteroids: a promising option in deciphering remedial strategies for abiotic stress tolerance in rice. *Front. Plant Sci.* 8:2151. 10.3389/fpls.2017.02151 29326745 PMC5742319

[B55] Siddiqui H. Hayat S. Bajguz A. (2018). Regulation of photosynthesis by brassinosteroids in plants. *Acta Physiol. Plant.* 40:59 10.1007/s11738-018-2639-2

[B56] Soshinkova T. N. Radyukina N. L. Korolkova D. V. Nosov A. V. (2013). Proline and functioning of the antioxidant system in *Thellungiella salsuginea* plants and cultured cells subjected to oxidative stress. *Russ. J. Plant Physiol.* 60 41–54. 10.1134/S1021443713010093

[B57] Strasser R. J. Srivastava A. Tsimilli-Michael M. (2000). “The Fluorescence transient as a tool to characterize and screen photosynthetic samples,” in *Probing Photosynthesis: Mechanism, Regulation and Adaptation*, eds Yunus M. Pathre U. Mohanty P. (New York, NY: CRC), 445–483.

[B58] Strasser R. J. Tsimilli-Michael M. Srivastava A. (2004). “Analysis of the chlorophyll a fluorescence transient,” in *Chlorophyll a Fluorescence: A Signature of Photosynthesis*, eds Papageorgiou G. C. Govinjee (Dordrecht: Springer), 321–362. 10.1007/978-1-4020-3218-9_12

[B59] Strizhov N. Abrahám E. Okrész L. Blickling S. Zilberstein A. Schell J. (1997). Differential expression of two P5CS genes controlling proline accumulation during salt-stress requires ABA and is regulated by ABA1, ABI1 and AXR2 in *Arabidopsis*. *Plant J.* 12 557–569. 10.1046/j.1365-313X.1997.00557.x 9351242

[B60] Surender Reddy P. Jogeswar G. Rasineni G. K. Maheswari M. Reddy A. R. Varshney R. K. (2015). Proline over-accumulation alleviates salt stress and protects photosynthetic and antioxidant enzyme activities in transgenic sorghum [*Sorghum bicolor* (L.) Moench]. *Plant Physiol. Biochem.* 94 104–113. 10.1016/j.plaphy.2015.05.014 26065619

[B61] Tani T. Sobajima H. Okada K. Chujo T. Arimura S. I. Tsutsumi N. (2008). Identification of the OsOPR7 gene encoding 12-oxophytodienoate reductase involved in the biosynthesis of jasmonic acid in rice. *Planta* 227 517–526. 10.1007/s00425-007-0635-7 17938955

[B62] Tomek P. Lazár D. Ilík P. Naus J. (2001). On the intermediate steps between the O and P steps in chlorophyll a fluorescence rise measured at different intensities of exciting light. *Funct. Plant Biol.* 28 1151–1160. 10.1071/PP01065

[B63] Venkatesh J. Upadhyaya C. P. Yu J.-W. Hemavathi A. Kim D. H. Strasser R. J. (2012). Chlorophyll a fluorescence transient analysis of transgenic potato overexpressing D-galacturonic acid reductase gene for salinity stress tolerance. *Hortic. Environ. Biotechnol.* 53 320–328. 10.1007/s13580-012-0035-1

[B64] Verma V. Ravindran P. Kumar P. P. (2016). Plant hormone-mediated regulation of stress responses. *BMC Plant Biol.* 16:86. 10.1186/s12870-016-0771-y 27079791 PMC4831116

[B65] Verslues P. E. Bray E. A. (2006). Role of abscisic acid (ABA) and *Arabidopsis thaliana* ABA-insensitive loci in low water potential-induced ABA and proline accumulation. *J. Exp. Bot.* 57 201–212. 10.1093/jxb/erj026 16339784

[B66] Wang Y. Mopper S. Hasenstein K. H. (2001). Effects of salinity on endogenous ABA, IAA, JA, and SA in *Iris hexagona*. *J. Chem. Ecol.* 27 327–342. 10.1023/A:1005632506230 14768818

[B67] Wasternack C. Strnad M. (2016). Jasmonate signaling in plant stress responses and development - active and inactive compounds. *N. Biotechnol.* 33 604–613. 10.1016/j.nbt.2015.11.001 26581489

[B68] Witzel K. Matros A. Strickert M. Kaspar S. Peukert M. Mühling K. H. (2014). Salinity stress in roots of contrasting barley genotypes reveals time-distinct and genotype-specific patterns for defined proteins. *Mol. Plant* 7 336–355. 10.1093/mp/sst063 24004485

[B69] Yusuf M. A. Kumar D. Rajwanshi R. Strasser R. J. Tsimilli-Michael M. Govindjee (2010). Overexpression of g-tocopherol methyl transferase gene in transgenic *Brassica juncea* plants alleviates abiotic stress: physiological and chlorophyll a fluorescence measurements. *Biochim. Biophys. Acta* 1797 1428–1438. 10.1016/j.bbabio.2010.02.002 20144585

[B70] Zeng H. Tang Q. Hua X. (2010). Arabidopsis brassinosteroid mutants det2-1 and bin2-1 display altered salt tolerance. *J. Plant Growth Regul.* 29 44–52. 10.1007/s00344-009-9111-x

[B71] Zhang X. Lu G. Long W. Zou Y. Li F. Nishio T. (2014). Recent progress in drought and salt tolerance studies in Brassica crops. *Breed. Sci.* 64 60–73. 10.1270/jsbbs.64.60 24987291 PMC4031111

[B72] Zhu M. Zhou M. Shabala L. Shabala S. (2017). Physiological and molecular mechanisms mediating xylem Na^+^ loading in barley in the context of salinity stress tolerance. *Plant Cell Environ.* 40 1009–1020. 10.1111/pce.12727 26881809

[B73] Żurek G. Rybka K. Pogrzeba M. Krzyżak J. Prokopiuk K. (2014). Chlorophyll a fluorescence in evaluation of the effect of heavy metal soil contamination on Perennial grasses. *PLoS One* 9:e91475. 10.1371/journal.pone.0091475 24633293 PMC3954697

[B74] Zushi K. Matsuzoe N. (2017). Using of chlorophyll a fluorescence OJIP transients for sensing salt stress in the leaves and fruits of tomato. *Sci. Hortic.* 219 216–221. 10.1016/j.scienta.2017.03.016

